# Effects of biological sex and oral contraceptive pill use on cutaneous microvascular endothelial function and nitric oxide-dependent vasodilation in humans

**DOI:** 10.1152/japplphysiol.00586.2022

**Published:** 2023-03-02

**Authors:** Casey G. Turner, Anna E. Stanhewicz, Karen E. Nielsen, Jeffrey S. Otis, Rafaela G. Feresin, Brett J. Wong

**Affiliations:** ^1^Department of Kinesiology and Health, Georgia State University, Atlanta, Georgia, United States; ^2^Department of Health and Human Physiology, University of Iowa, Iowa City, Iowa, United States; ^3^Department of Population Health Sciences, School of Public Health, Georgia State University, Atlanta, Georgia, United States; ^4^Department of Nutrition, Georgia State University, Atlanta, Georgia, United States

**Keywords:** endothelium, microdialysis, nitric oxide, women

## Abstract

The purpose of this study was to evaluate in vivo endothelial function and nitric oxide (NO)-dependent vasodilation between women in either menstrual or placebo pill phases of their respective hormonal exposure [either naturally cycling (NC) or using oral contraceptive pills (OCPs)] and men. A planned subgroup analysis was then completed to assess endothelial function and NO-dependent vasodilation between NC women, women using OCP, and men. Endothelium-dependent and NO-dependent vasodilation were assessed in the cutaneous microvasculature using laser-Doppler flowmetry, a rapid local heating protocol (39°C, 0.1 °C/s), and pharmacological perfusion through intradermal microdialysis fibers. Data are represented as means ± standard deviation. Men displayed greater endothelium-dependent vasodilation (plateau, men: 71 ± 16 vs. women: 52 ± 20%CVC_max_, *P* < 0.01), but lower NO-dependent vasodilation (men: 52 ± 11 vs. women: 63 ± 17%NO, *P* = 0.05) compared with all women. Subgroup analysis revealed NC women had lower endothelium-dependent vasodilation (plateau, NC women: 48 ± 21%CVC_max_, *P* = 0.01) but similar NO-dependent vasodilation (NC women: 52 ± 14%NO, *P* > 0.99), compared with men. Endothelium-dependent vasodilation did not differ between women using OCP and men (*P* = 0.12) or NC women (*P* = 0.64), but NO-dependent vasodilation was significantly greater in women using OCP (74 ± 11%NO) than both NC women and men (*P* < 0.01 for both). This study highlights the importance of directly quantifying NO-dependent vasodilation in cutaneous microvascular studies. This study also provides important implications for experimental design and data interpretation.

**NEW & NOTEWORTHY** This study supports differences in microvascular endothelial function and nitric oxide (NO)-dependent vasodilation between women in low hormone phases of two hormonal exposures and men. However, when separated into subgroups of hormonal exposure, women during placebo pills of oral contraceptive pill (OCP) use have greater NO-dependent vasodilation than naturally cycling women in their menstrual phase and men. These data improve knowledge of sex differences and the effect of OCP use on microvascular endothelial function.

## Graphical abstract

**Figure:**
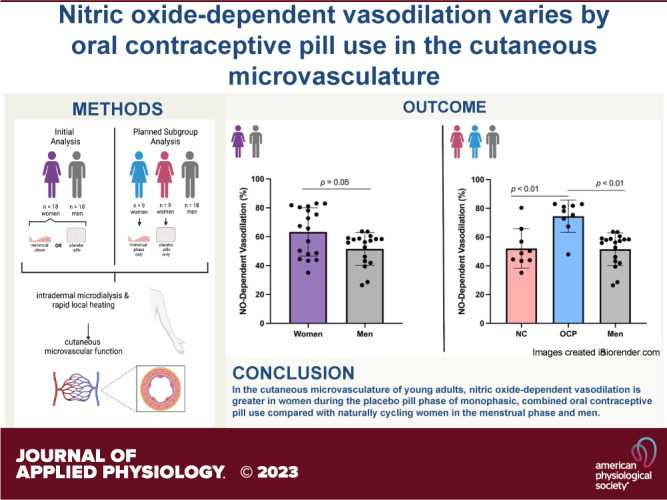

## INTRODUCTION

Microvascular function plays an important role in blood pressure and blood glucose regulation, by influencing total peripheral resistance and glucose uptake, respectively (1). Cutaneous microvascular function is representative of systemic microvascular function (2), is easily accessible, and yields reproducible results (2); however, basic physiology of sex differences in cutaneous microvascular responses remains incompletely understood. To date, a limited number of studies have directly compared cutaneous microvascular function between young men and women (3–7). A smaller number of studies have investigated this question in response to rapid local heating (3–5), which is a common stimulus to induce microvascular vasodilation, in part due to a large reliance on nitric oxide (NO) (8, 9). These studies suggest similar microvascular endothelium-dependent vasodilation between men and women (3–5) but possible differences in contributing mechanisms, specifically NO (4, 5). Additional investigation of sex differences in cutaneous microvascular function in response to local heating is warranted for experimental design, data interpretation, and translation of findings.

In vascular research, premenopausal women are often restricted to experimental testing during low hormone phases, because female sex hormones may impact vascular function (10, 11). During the natural menstrual cycle, testing is often completed during the menstrual/early follicular (M/EF) phase (*days 1–7*), as this corresponds to the lowest circulating levels of 17β-estradiol (E2) and progesterone. Premenopausal women in the low hormone phase of oral contraceptive pill (OCP) use (i.e., placebo pills) are often grouped together with naturally cycling (NC) women as an assumed homogenous group, as circulating concentrations of both endogenous and exogenous hormones should be low in these women during this time. However, it remains unclear if vascular function is similar between women in these hormonal exposures, especially in the microvasculature. Previous work has investigated this question in large arteries via brachial artery flow-mediated dilation (12), but large vessel function may not accurately reflect microvascular function (13, 14). Furthermore, in the cutaneous microvasculature, when a study cohort included only NC women, NO-dependent vasodilation was lower in women compared with men (4); however, when a study cohort included NC women and women using OCP, NO-dependent vasodilation in women did not differ from men (5). This suggests that NO-dependent vasodilation may differ between women using OCP and NC women, regardless of testing during respective low hormone phases.

The primary purpose of this study was to investigate cutaneous microvascular endothelium-dependent and NO-dependent vasodilation between women in low hormone phases of their respective hormonal exposure (either NC or using OCP) and men. A secondary purpose of this study was to compare cutaneous microvascular endothelium-dependent and NO-dependent vasodilation between subgroups of NC women, women using OCP, and men, in a planned subgroup analysis. We hypothesized that *1*) the magnitude of endothelium-dependent and NO-dependent vasodilation would be similar between all women and men, *2*) endothelium-dependent vasodilation would be similar between the two subgroups of women (i.e., NC women and women using OCP), and *3*) NO-dependent vasodilation would be greater in women using OCP compared with NC women in their respective low hormone phases. An exploratory aim was also included to assess sex differences in cutaneous sensory nerve-mediated vasodilation, which can be assessed using the same methodology (3, 5, 9, 15, 16), for which we hypothesized that sensory nerve-mediated vasodilation would be *1*) similar between all women and men and *2*) similar between the two subgroups of women.

## METHODS

### Ethical Approval

All protocols and procedures were approved by Advarra Institutional Review Board (Columbia, MD; No. Pro00024265, No. Pro00056105) and the United States Food and Drug Administration (IND 138231, IND 157532). All experimental procedures conformed with the Declaration of Helsinki. Each participant provided written and verbal consent before participating in any experimental procedure.

### Participants

Participants included men (*n* = 18) and women (*n* = 18 total) who were either NC (*n* = 9) or using OCP (*n* = 9). All women using OCPs were taking monophasic, combination formulations for at least 3 mo. The average duration of OCP use was 30 ± 32 mo (range: 3–108 mo). Further details about the OCPs participants were using, including components, dosages, and generation, are shown in Table 1. Women were tested during commonly used windows to represent low hormone phases [NC, *days 2–5* of natural menstrual cycle; OCP, *days 1–2* of placebo pills (3–7, 17)], where circulating endogenous (e.g., NC) or exogenous (e.g., OCP) hormones should be low. NC women were tested on *day 2* (*n* = 2), *day 3* (*n* = 3), *day 4* (*n* = 3), or *day 5* (*n* = 1) of the natural menstrual cycle. Women using OCP were tested on *day 1* (*n* = 3) or *day 2* (*n* = 6) of placebo pills. Phase was determined by self-report and confirmed via self-report cycle tracking or presentation of OCP pack. Plasma E2 was also assessed. All women were required to submit a urine pregnancy test (McKesson hCG Combo Test Cassette, Consult Diagnostics; Richmond, VA) to confirm negative pregnancy status. Participants were recruited and included to achieve a similar proportion of White and non-White young adults within each group due to previous reports of an effect of racial identity on cutaneous microvascular function (5, 18–21).

**Table 1.** Oral contraceptive pill details: included components, dosages, and generation

Number of Participants	EE Dose	Progestin Dose	Generation
2	0.01 mg EE	1 mg Norethindrone acetate	1st
2	0.02 mg EE	1 mg Norethindrone acetate	1st
1	0.035 mg EE	0.25 mg Norgestimate	2nd
1	0.03 mg EE	0.15 mg Desogestrel	3rd
2	0.02 mg EE	3 mg Drospirenone	4th
1	0.03 mg EE	3 mg Drospirenone	4th

EE, ethinyl estradiol.

Participants were young adults (< 40 yr old, range: 18–36 yr for the entire cohort) to mitigate potential age-related declines in NO-dependent vasodilation (22) and circulating female sex hormones (23) and normotensive (systolic blood pressure < 120 mmHg and diastolic blood pressure < 80 mmHg). Self-report health history was obtained, and all participants were free of cardiovascular, pulmonary, and metabolic diseases and had no history of nerve pain/damage, cancer (chemotherapy or radiation therapy), or skin disorders (e.g., psoriasis). No participants used tobacco products, nicotine products, supplements, or medications (except women using OCPs). Further exclusion criteria included body mass index (BMI) > 30 kg/m^2^ that was clearly due to excessive adiposity, active COVID-19 infection, <1-mo postknown COVID-19 infection, or long-lasting symptoms after known COVID-19 infection (24). All participants reported engaging in moderate physical activity at least 3 days/wk. Participants were asked to refrain from alcohol, vigorous exercise, caffeine, and high-fat meals for at least 8 h before the experimental protocol.

### Instrumentation

Participants were seated in the semirecumbent position. The experimental arm (left) was positioned and secured at heart level to minimize the effect of hydrostatic pressure on blood flow. Skin blood flow data were analyzed at one intradermal microdialysis fiber site (CMA 31; Harvard Apparatus, Hollister, MA) on the dorsal forearm. An ice pack was used to numb the skin (25), and a 23-gauge needle was used to make an entry and exit point in the dermal layer of the skin. The microdialysis fiber was threaded through the lumen of the needle, the microdialysis membrane was left in the dermal layer, and the needle was removed.

To control local skin temperature, a local heater unit (VHP1 heater units and VMS-HEAT controller; Moor Instruments, Axminster, UK) was placed directly over the microdialysis membrane. An integrated laser-Doppler probe (VP7b probes and VMS-LDF2 monitor; Moor Instruments) was placed in the center of the local heating unit to obtain red blood cell (RBC) flux, an index of skin blood flow, at the microdialysis site. Blood pressure was measured every 10 min from the contralateral arm (right) using an automated brachial oscillometric device, and heart rate was derived from pulse detection (Welch Allyn Vital Signs Series 6000; Skaneateles Falls, NY). Mean arterial pressure (MAP) was calculated as one-third pulse pressure plus diastolic pressure.

### Experimental Protocol

Women participants were asked to supply a venous blood sample to assess plasma levels of E2. Blood samples were obtained by a certified phlebotomist or registered nurse via basic venipuncture and using aseptic protocols. Approximately 20 mL of blood was collected in EDTA vacutainers. Samples were centrifuged at 1,000 RPM, plasma was aliquoted into microtubules, and plasma was stored at −80°C until analyzed (estradiol ELISA kit, Product No. 501890, Cayman Chemical, Ann Arbor, MI).

The microdialysis site was perfused with lactated Ringer solution (Baxter Healthcare, Deerfield, IL) through a trauma resolution period after microdialysis fiber placement (∼45–60 min), baseline data collection, and a rapid local heating protocol. The local heater unit was first set to thermoneutral temperature (33°C), and baseline skin blood flow was assessed for ∼15 min. After the baseline measurement, a rapid local heating protocol was conducted to elicit endothelium-dependent vasodilation, where local heater temperature was increased to 39°C at a rate of 0.1°C/s (8). No participants reported pain sensations during the local heating protocol. Once a plateau in skin blood flow was achieved (∼30–40 min into local heating), 20 mM *N*^ω^-nitro-l-arginine methyl ester (l-NAME), a nonspecific NO synthase (NOS) inhibitor, was perfused through the microdialysis fiber to assess the contribution of NO to vasodilation (9, 18, 19, 26–28). Once a new plateau after l-NAME perfusion (i.e., post-l-NAME plateau) was achieved (∼30 min into l-NAME infusion), maximal vasodilation was induced by heating the skin to 43°C (0.1°C/s) and infusing 28 mM sodium nitroprusside (29). All solutions were perfused at a rate of 2 μL/min (Beehive Controller and Baby Bee syringe pumps; Bioanalytical Systems, West Lafayette, IN). Pharmacological agents were diluted with sterile lactated Ringer solution (30) and drawn through filter needles (BD Filter Needle; Becton Dickinson, Franklin Lakes, NJ).

### Data Analysis

Skin blood flow data (RBC flux) were continuously recorded at 40 Hz using commercially available hardware and software (PowerLab 16/35 data acquisition and LabChart 8 software; ADInstruments, Colorado Springs, CO). Cutaneous vascular conductance (CVC) was calculated as RBC flux divided by MAP and standardized to site-specific maximal vasodilation (%CVC_max_). Five main periods of skin blood flow data were analyzed: *1*) baseline, *2*) initial peak [i.e., sensory nerve-mediated vasodilation (9, 15, 16)], *3*) plateau (i.e., endothelium-dependent vasodilation (8, 9, 31)), *4*) post-l-NAME plateau (used to calculate NO-dependent vasodilation), and *5*) maximal vasodilation. The initial peak is a rapid, but transient, phase, and thus ∼60 s of data were analyzed for this period. The following 3-min windows of data were analyzed for the remaining four phases: immediately preceding the onset of the local heating protocol for baseline, immediately preceding the infusion of l-NAME for the plateau, immediately preceding initiation of maximal vasodilation for the post-l-NAME plateau, and before the cessation of the experimental protocol for maximal vasodilation. NO-dependent vasodilation (%NO) was calculated as the percent change from plateau to the post-l-NAME plateau (27).

### Statistical Analysis

We completed an initial analysis comparing women and men, followed by a planned subgroup analysis. Primary outcome variables included plateau and calculated NO-dependent vasodilation and were, therefore, used to power these analyses. Sample size for both analyses was determined with a priori power analysis. Effect size was specified based on preliminary data collected in our laboratory. Our initial preliminary data suggested a large effect size (*d* = 1.4) of the mean difference in plateau between women (either NC or using OCP) in their respective low hormone phases and men (+18%CVC_max_ in men vs. women). Assuming an α level of 0.05 and 95% power, this resulted in a sample size of *n* = 14 per group. To protect against potential underestimation of the standard deviation (SD) in the preliminary data, we increased the sample size by 25%, to result in a final sample size of *n* = 18 per group. Sample size estimation for the subgroup analysis was based on preliminary data for NO-dependent vasodilation between subgroups of women (NC women vs. women using OCP). Our preliminary data suggested a large effect size (*d* = 2.3) of the mean difference in NO-dependent vasodilation between NC women during the menstrual phase and women using OCP during the placebo pill phase (−31%NO in NC women vs. women using OCP). Assuming an α level of 0.05 and 95% power, this resulted in a sample size of *n* = 7 per group. Sample size was, again, increased by 25% in case of underestimation of the SD, to result in a final sample size of *n* = 9 per group. Secondary outcome variables included baseline, initial peak, post-l-NAME plateau, and maximal skin blood flow.

All outcome measures were tested for equal variance between groups (Levene’s test) and normality (Shapiro–Wilk test) before analysis. Variance was not statistically different in any comparison. Only baseline, plateau, and post-l-NAME plateau data were determined to be normally distributed overall and within subgroups. Therefore, parametric statistical tests were used to analyze baseline, plateau, and post-l-NAME data (independent samples *t* test, one-way ANOVA), and nonparametric statistical tests were used to analyze maximal, initial peak, and %NO data (Mann–Whitney *U* test, Kruskal–Wallis test). Furthermore, in the subgroup analysis, Tukey’s correction factors were used to account for multiple pairwise comparisons for one-way ANOVA and Dunn’s correction factors for Kruskal–Wallis tests. In addition, an independent samples *t* test was used to compare plasma E2 between NC and OCP women, and a preliminary Pearson’s correlation was used to assess the potential correlation between duration of OCP use and plateau or %NO-dependent vasodilation in women using OCP. The level of significance was set at α ≤ 0.05 for all statistical tests. All data are presented as means ± SD with 95% confidence intervals (CI), and all data were analyzed and graphed using commercially available software (SAS, Cary, NC and GraphPad Prism 8, San Diego, CA).

## RESULTS

### Participant Hemodynamics

Participant demographic and hemodynamic information is shown in Table 2. There was no statistical difference in measured E2 between subgroups of women (NC women: 116 ± 106 pg/mL, OCP women: 82 ± 61 pg/mL, *P* = 0.50).

**Table 2.** Participant characteristics

	Women (*n* = 18)		Men (*n* = 18)
	NC Women	OCP Women	
	(*n* = 9)	(*n* = 9)	
Age, yr	22 ± 3	22 ± 3	25 ± 6
Self-identified race/ethnicity	5 NHB, 4 NHW	2 H, 3 NHB, 4 NHW	10 NHB, 8 NHW
Height, m	1.64 ± 0.08	1.68 ± 0.06	1.78 ± 0.09
Mass, kg	71.5 ± 22.2	70.0 ± 10.7	77.8 ± 12.7
Body mass index (BMI) kg/m^2^	25 ± 6	25 ± 3	25 ± 3
Resting heart rate, beats/min	69 ± 6	71 ± 6	60 ± 7
Systolic blood pressure, mmHg	111 ± 7	115 ± 3	116 ± 5
Diastolic blood pressure, mmHg	72 ± 5	74 ± 3	70 ± 5
Mean arterial pressure, mmHg	85 ± 5	87 ± 3	85 ± 4

Data are presented as means ± SD. H, Hispanic; NC, naturally cycling; NHB, non-Hispanic Black; NHW, non-Hispanic White; OCP, oral contraceptive pills.

### All Women versus Men

**Figure 1.**
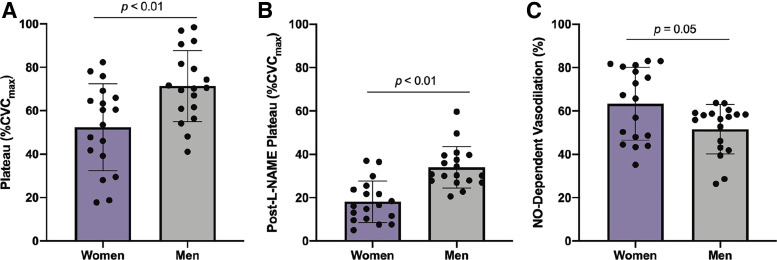
Responses to local heating between women and men. *A*: plateau. *B*: post-l-NAME plateau. *C*: NO-dependent vasodilation. Purple bars, all women (*n* = 18). Gray bars, men (*n* = 18). Data are represented as means ± SD. NO-dependent vasodilation data was analyzed with Mann–Whitney test. Plateau and post-l-NAME plateau data were analyzed with independent samples *t* tests. Level of significance was set at 0.05. Statistical significance is indicated as a horizontal line over respective group bars and labeled with the *P* value of the comparison. %CVCmax, percent of maximal cutaneous vascular conductance; l-NAME, N^ω^-nitro-l-arginine methyl ester; NO, nitric oxide.

Maximal CVC and baseline (%CVC_max_) data for the complete cohort is shown in Table 3. There were no statistically significant differences between groups in maximal CVC; however, baseline was greater in men than women (Table 3). Figure 1, *A–C* shows data comparing all women and men. The magnitude of the plateau (Fig. 1*A*) was greater in men (71 ± 16%CVC_max_; 95% CI: 63%–80%CVC_max_) than in women (52 ± 20%CVC_max_; 95% CI: 43%–62%CVC_max_; *P* < 0.01). The post-l-NAME plateau (Fig. 1*B*) was greater in men (34 ± 10%CVC_max_; 95% CI: 29%–39%CVC_max_) than in women (18 ± 10%CVC_max_; 95% CI: 13%–23%CVC_max_; *P* < 0.01), suggesting men have greater absolute NO-independent dilation in response to local heating than women. NO-dependent vasodilation (Fig. 1*C*) was lower in men (52 ± 11%NO; 95% CI: 46%–57%NO) compared with women (63 ± 17%NO; 95% CI: 55%–72%NO; *P* = 0.05).

**Table 3.** Maximal and baseline skin blood flow in women vs. men

	Women	Men	
	(*n* = 18)	(*n* = 18)	*P* Value
Maximal blood flow, flux/mmHg	2.67 ± 0.68	2.42 ± 0.85	0.10
Baseline blood flow, %CVC_max_	12 ± 5	17 ± 7	0.01

Data are represented as means ± SD. Maximal blood flow was analyzed with a Mann–Whitney test, and baseline blood flow was analyzed with an independent samples *t* test. Statistical significance was set at 0.05. %CVC_max_, percent of maximal cutaneous vascular conductance.

### Subgroup Analysis

**Figure 2.**
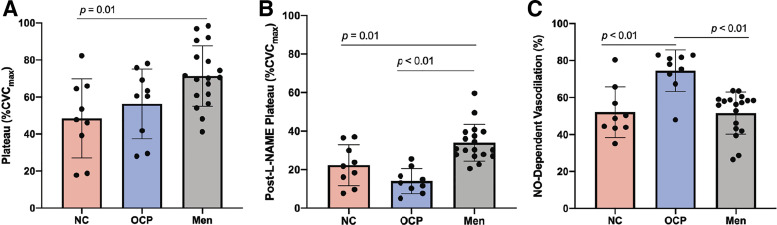
Responses to local heating between NC women, women using OCP, and men. *A*: plateau. *B*: post-l-NAME plateau. *C*: NO-dependent vasodilation. Pink bars, NC women (*n* = 9). Blue bars, women using OCP (*n* = 9). Gray bars, men (*n* = 18). Data are represented as means ± SD. NO-dependent vasodilation data was analyzed with Kruskal–Wallis test. Plateau and post-l-NAME plateau data were analyzed with one-way ANOVAs. Tukey’s correction factors were used to account for multiple pairwise comparisons for one-way ANOVAs and Dunn’s correction factors for Kruskal–Wallis test. Level of significance was set at 0.05. Statistical significance is indicated as a horizontal line over respective group bars and labeled with the *P* value of the comparison. l-NAME, N^ω^-nitro-l-arginine methyl ester; NC, naturally cycling; NO, nitric oxide; OCP, oral contraceptive pill.

Maximal CVC (*P* = 0.10) and baseline (*P* = 0.05, but no significant post hoc comparisons) between subgroups are shown in Table 4. There were no statistically significant differences in maximal CVC or baseline blood flow between subgroups (Table 4). Figure 2, *A–C* shows data between NC women, OCP women, and men. The plateau was 48 ± 21%CVC_max_ in NC women (95% CI: 32%–65%CVC_max_), 56 ± 19%CVC_max_ in women using OCP (95% CI: 42%–71%CVC_max_), and 71 ± 16%CVC_max_ in men (Fig. 2*A*). There was a statistically significant difference between groups for plateau (*P* = 0.01), where plateau was greater in men compared with NC women (*P* = 0.01). There was no statistically significant difference in plateau between women using OCP and men (*P* = 0.12) or NC women (*P* = 0.64). The post-l-NAME plateau was 22 ± 11%CVC_max_ in NC women (95% CI: 14%–31%CVC_max_), 14 ± 7%CVC_max_ in women using OCP (95% CI: 9%–19%CVC_max_), and 34 ± 10%CVC_max_ in men (Fig. 2*B*). There was a statistically significant difference between groups for post-l-NAME plateau (*P* < 0.01), where men displayed a greater magnitude of post-l-NAME plateau compared with NC women (*P* = 0.01) and women using OCP (*P* < 0.01), again suggesting men have greater absolute NO-independent dilation in response to local heating compared with both subgroups of women. There was no statistically significant difference for post-l-NAME plateau between subgroups of women (*P* = 0.15). NO-dependent vasodilation was 52 ± 14%NO in NC women (95% CI: 42%–63%NO), 74 ± 11%NO in women using OCP (95% CI: 66%–83%NO), and 52 ± 11%NO in men (Fig. 2*C*). There was a statistically significant difference between groups for NO-dependent vasodilation (*P* < 0.01), where women using OCP displayed greater %NO compared with NC women (*P* < 0.01) and men (*P* < 0.01). There was no statistically significant difference in NO-dependent vasodilation between men and NC women (*P* > 0.99). Furthermore, for women using OCP, there was no significant correlation between duration of OCP use and endothelium-dependent vasodilation (plateau; *r* = −0.30, *P* = 0.47) or NO-dependent vasodilation (*r* = 0.00, *P* > 0.99; data not shown).

**Table 4.** Maximal and baseline skin blood flow in 3 subgroups

	NC Women	Women Using OCP	Men
	(*n* = 9)	(*n* = 9)	(*n* = 18)
Maximal blood flow, flux/mmHg	2.44 ± 0.55	2.91 ± 0.75	2.42 ± 0.85
Baseline blood flow, %CVC_max_	11 ± 6	12 ± 3	17 ± 7

Data are represented as means ± SD. Maximal blood flow was analyzed with a Kruskal–Wallis test, and baseline blood flow was analyzed with one-way ANOVA. Statistical significance was set at 0.05. %CVC_max_, percent of maximal cutaneous vascular conductance; NC, naturally cycling; OCP, oral contraceptive pill.

### Sensory Nerve-Mediated Vasodilation

**Figure 3.**
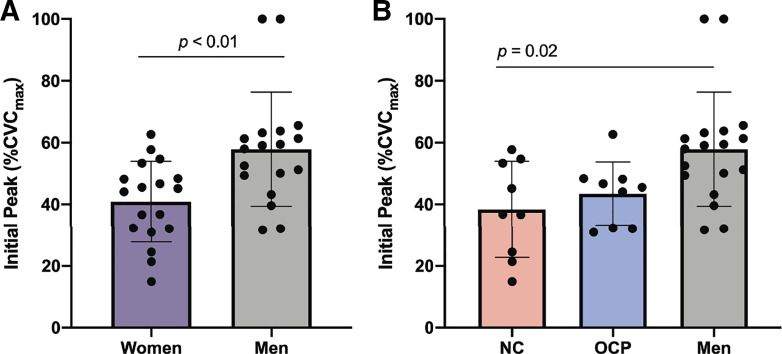
Sensory nerve-mediated vasodilation in response to local heating. *A*: initial peak in men vs. women. Purple bars, all women (*n* = 18). Gray bars, men (*n* = 18). Data was analyzed with Mann–Whitney test. *B*: initial peak between subgroups. Pink bars, NC women (*n* = 9). Blue bars, women using OCP (*n* = 9). Gray bars, men (*n* = 18). Data was analyzed with Kruskal–Wallis test. Dunn’s correction factors were used to account for multiple pairwise comparisons for Kruskal–Wallis tests. All data are represented as means ± SD. Level of significance was set at 0.05. Statistical significance is indicated as a horizontal line over respective group bars and labeled with the *P* value of the comparison. %CVCmax, percent of maximal cutaneous vascular conductance; l-NAME, N^ω^-nitro-l-arginine methyl ester; NC, naturally cycling; NO, nitric oxide; OCP, oral contraceptive pills.

Figure 3 shows results addressing the exploratory aim to assess sensory nerve-mediated vasodilation in this cohort. The magnitude of the initial peak (Fig. 3*A*) was greater in men (58 ± 18%CVC_max_; 95% CI: 49%–67%CVC_max_) than all women (41 ± 13%CVC_max_; 95% CI: 34%–47%CVC_max_; *P* < 0.01). When divided into subgroups, the initial peak was 38 ± 16%CVC_max_ in NC women (95% CI: 26%–50%CVC_max_), 43 ± 10%CVC_max_ in women using OCP (95% CI: 36%–51%CVC_max_), and 58 ± 18%CVC_max_ in men (Fig. 3*B*). There was a statistically significant difference between groups for initial peak (*P* = 0.01), where initial peak was greater in men than NC women (*P* = 0.02). There was no statistically significant difference in initial peak between women using OCP and men (*P* = 0.08) or NC women (*P* > 0.99).

## DISCUSSION

There are two key findings from this study. First, in contrast with our hypothesis, there are sex differences in microvascular responses to rapid local heating of the skin to 39°C when women are tested during the menstrual phase of the natural menstrual cycle or during placebo phase of OCP. Second, in agreement with our hypothesis, NO-dependent vasodilation is greater in women during the placebo pill phase of OCP use compared with both NC women in the menstrual phase of the natural menstrual cycle and men. These findings address current gaps in the literature that are pertinent to experimental design, data interpretation, and translation of findings.

### Overall Sex Differences

In this sample of women and men, there are sex differences in microvascular responses to rapid local heating of the skin to 39°C. We observed a greater magnitude of plateau in men compared with women in low hormone phases, suggesting greater endothelium-dependent vasodilation (8, 9, 31) in men. In contrast with this study, previous studies suggest no sex differences in plateau when heating to 39°C (5) or 42°C (3, 4). Although end temperatures of both 39°C and 42°C represent submaximal thermal stimuli, local heating to 39°C elicits ∼50%CVC_max_ and 42°C elicits near-maximal vasodilation (∼85%–95%CVC_max_) in the skin (32). Therefore, when heating to 42°C, near-maximal vasodilation responses may be similar between sexes, but local heating to lower thermal stimuli may reveal physiological sex differences. The present data indirectly supports this with no observed difference in maximal CVC between groups (Tables 3 and 4). Other reports suggest there is no sex difference in the endothelium-dependent or endothelium-independent reactivity to vasodilator pharmacological stimuli in the cutaneous microvasculature, further suggesting that methodology and specific stimuli may be important considerations for interpretation of findings (7). However, we did observe greater baseline blood flow in men compared with women in our sample (Table 3). This agrees with a previous report of greater sympathetic regulation of basal blood flow in women compared with men (33). Therefore, women may have to overcome an initial withdrawal of sympathetic regulation that men do not and may, thus, yield a lower response to a submaximal stimulus, such as heating the skin to 39°C.

Previous data suggest greater cutaneous microvascular vasodilation in White young adults compared with young adults who identify as Black or African American (5, 18–21). Therefore, it is possible the effect of biological sex observed in this study may have been influenced by racial identity/ethnicity of the participants in each group, but we were not powered to assess the effect of race/ethnicity. However, given that we recruited a similar proportion of White and non-White young adults within each subgroup, any effect due to race/ethnicity would be balanced across groups and would be unlikely to be a major explanation for our current findings.

Although men exhibited a greater magnitude of the plateau to local heating than women, the magnitude of the post-l-NAME plateau was also greater in men than in women, resulting in lower NO-dependent vasodilation in men versus women. Previous reports suggest greater endothelial NOS [eNOS, the protein responsible for NO production in the skin in response to local heating (31)] expression and mRNA in female compared with male endothelial cells (34), but it is currently unclear how eNOS expression may differ between men and women within the cutaneous microvasculature. In the present study, the pattern of the local heating response observed in men relative to women suggests a greater dependence on NO-independent mechanisms, such as endothelium-derived hyperpolarizing factors (EDHF) (35), in men compared with women in low hormone phases. The present study was not designed to examine NO-independent mechanisms, and this may therefore warrant further investigation.

### Hormonal Exposures in Women

The subgroup analysis revealed that differences in plateau between all women and men were largely driven by NC women (Fig. 2*A*), whereas differences in NO-dependent vasodilation between all women and men were largely driven by women using OCP (Fig. 2*C*). These findings suggest that inclusion of both NC women and women using OCP in the same sample may minimize the ability to accurately interpret cutaneous microvascular function data for sex differences, regardless of testing women during respective low hormone phases. For instance, when considering all women compared with men, the data indicated significantly greater NO-dependent vasodilation in women (63 ± 17%NO) compared with men (52 ± 11%NO, Fig. 1*C*). However, during the subgroup analysis, NO-dependent vasodilation between NC women during the menstrual phase and men was nearly identical within our cohort (NC women, 52 ± 14%NO, Fig. 2*C*). These findings are important for experimental design, as the inclusion of women in both hormonal exposures (in total, as well as in proportion to each other) may confound results or yield studies that are not reproducible.

Present findings also indicate that NO-dependent vasodilation differs between NC women and women using OCP during their respective low hormone phases (NC women: 52%NO, OCP women: 74%NO; Fig. 2*C*). Although this study does not provide insight into the mechanism(s) underlying this difference in NO-dependent vasodilation, women using OCP have previously been shown to have increased eNOS mRNA expression within the skin compared with NC women (36). There is similar evidence suggesting an increased reliance on NO in women using OCP compared with NC women, such that women using OCP showed increased vasoconstrictor responses to NOS inhibition (37). However, this increase in eNOS expression may not coincide with increases in vasodilation responses (36). This may be related to other reports of increased renin-angiotensin-aldosterone system activity, oxidative stress, and inflammation in women using OCP (36, 38–41) that may counteract or blunt NO effects on vascular function. Future studies assessing underlying mechanisms and these responses during high hormone exposure may provide additional insight into this question.

The NO contribution was the only portion of the response for which we found a statistically significant difference between subgroups of women in this analysis. The magnitude of endothelium-dependent vasodilation (i.e., plateau) was not statistically discernible between groups (NC women, 48%CVC_max_; women using OCP, 56%CVC_max_). This is analogous with previous data regarding brachial artery endothelium-dependent vasodilation between NC women and women using OCP (12). Though not statistically significant, there was a large effect size for the post-l-NAME plateau between groups of women (NC vs. OCP, mean difference, +8%CVC_max_, *d* = 0.9) within the present study. Collectively, the marginally greater plateau and lower post-l-NAME plateau contributed to a greater calculated NO-dependent vasodilation in women using OCP. These data underscore why it is important to quantify NO-dependent vasodilation, as solely measuring the magnitude of the plateau may not address the underlying contribution of NO. In addition, the finding of similar plateau values, yet different contributions from NO, between subgroups of women may indicate an increase in EDHF-mediated vasodilation in NC women during the menstrual phase. Although NO is implicated as the main contributor to thermal hyperemia in the skin, several pathways contribute to the response (5, 9, 18, 29, 35, 42, 43), and the contribution of mediating mechanisms has yet to be defined clearly in subgroups of women. Therefore, although grouping women in various hormonal exposures together increases the inclusion of women in research, it may hinder the ability to delineate effects of endogenous and exogenous hormones on vascular and endothelial function in women, which may have practical and/or clinical relevance.

Exogenous hormone exposure is a regular and common aspect of life for premenopausal women. Indeed, ∼28% of premenopausal women currently use some form of hormonal contraceptive method in the United States, with ∼14% attributable to OCP (44). Specific to the age range included in the present study, roughly 20% of women aged 15–19 yr and 20–29 yr use OCP whereas ∼11% aged 30–39 yr use OCP (44). Therefore, excluding all women using hormonal contraceptive methods from vascular research is also not an appropriate solution. We suggest that participant inclusion be designed with targeted research aims in mind. When research is aimed to assess population-based outcomes, it may be appropriate to include women in various hormonal states or phases, as this increases external validity. However, when research is aimed to assess between or within sex differences, especially regarding mechanistic pathways, hormonal exposures should be taken into consideration and inclusion should be planned accordingly.

### Sensory Nerve-Mediated Vasodilation

A greater magnitude of initial peak was observed in men compared with all women (Fig. 3*A*), suggesting greater sensory nerve-mediated (9, 15, 16) vasodilation in men. In contrast, previous studies suggest no sex differences in initial peak (3, 5). Again, the near-maximal stimulus of 42°C may result in similar sensory nerve-mediated responses in men and women, but local heating to lower thermal stimuli (such as 39°C) may reveal physiological sex differences.

### Limitations

Although the sample sizes in the present study sizes determined by a priori power analysis, evaluation within a larger sample may be warranted to confirm results and investigate potential mechanisms. Furthermore, criteria to determine experimental time frames for phasic testing in premenopausal women are inconsistent within the relevant literature. During OCP use, peak circulating concentrations of exogenous hormones are typically reached within 1–2 h of oral consumption, with molecules clearing the system within 10–24 h on average (45). Elevations in endogenous E2 and follicular maturation have been recorded toward the end of the placebo pill week of OCP use (46, 47), indicating later days within the placebo pill week may allow reawakening of the hypothalamus-pituitary-ovarian axis, accounting for rises in endogenous hormone production within 7 days. Therefore, *days 1* and *2* of placebo pills in the OCP monthly cycle, as used in the present study, should correspond to low circulating concentrations of exogenous, as well as endogenous, hormones. However, it may also be warranted to assess the phenomenon measured in the present study during other days of the placebo pill week of OCP use for confirmation over the entire placebo pill period.

Further limitations of this study are as follows. First, we did not perform blood analyses for fasting blood glucose or lipid profile. Therefore, we cannot objectively conclude that no participants had altered glucose or lipid status. However, all participants were normotensive and did not use any medications (except for women using OCP). Furthermore, no participants reported being diagnosed with prediabetes, type 1 or 2 diabetes, hypercholesterolemia, or dyslipidemia. Second, women did not undergo cycle tracking before experimental testing. However, the menstrual phase of the natural menstrual cycle is an easily self-tracked phase. All NC women verbally confirmed current day of cycle at experimental visits based on menstrual bleeding, which was, in all cases, confirmed by self-reported cycle tracking via mobile-based apps. Third, day/phase of the natural menstrual cycle or OCP use was self-reported. Therefore, these studies operated under the assumption that included women were honest about day/phase of their natural menstrual cycle or OCP use. In addition, experimental design relied on the assumption that women using OCP were compliant with their pill administration (i.e., not missing pills) and administered their pills/doses at consistent times across days. Fourth, women using any monophasic, combination OCP were allowed to participate in this study. It is common within the field of cutaneous microvascular research to include women using any variety of monophasic, combination OCP. Therefore, this choice in participant inclusion reflects this current practice within the field; however, investigation of the effect of other types of OCP or hormonal contraception methods on mechanisms of vascular function is warranted. Finally, this study did not employ an eNOS-specific inhibitor to quantify NO-dependent vasodilation. Therefore, it is not certain whether these results reflect eNOS function; however, previous studies suggest the eNOS isoform is largely responsible for NO production during local, nonpainful heating of the skin (31, 48, 49).

### Implications

These data highlight the importance of quantifying NO-dependent vasodilation, instead of inferring based on the overall magnitude of the plateau. Although NO may be a major contributor to the local heating response within the skin under many circumstances, it is not the sole known contributor, and an extensive breakdown of mediating mechanisms across subgroups within the populations has not been completed. Furthermore, when research is aimed to investigate underlying mechanisms of endothelial function or to deduce between or within sex differences, hormonal exposure of women participants should be considered, and inclusion should be planned carefully.

### Conclusions

The submaximal thermal stimulus of 39°C elicits a greater magnitude of endothelium-dependent vasodilation in men compared with women in the menstrual phase of the natural menstrual cycle or placebo pill phase of OCP use when grouped together. Cutaneous microvascular NO-dependent vasodilation is greater in women during the placebo pill phase of monophasic, combined OCP use compared with NC women in the menstrual phase and men. However, NO-dependent vasodilation is similar between men and NC women in the menstrual phase. Data from this study further the understanding of between and within sex differences in microvascular endothelial function, as well as the potential impact of OCP on mechanisms contributing to microvascular function, highlighting areas in need of further investigation.

## DATA AVAILABILITY

Data will be made available upon reasonable request.

## GRANTS

This study was funded by the National Heart, Lung, and Blood Institute Grant R01 HL141205 (to B.J.W.).

## DISCLOSURES

No conflicts of interest, financial or otherwise, are declared by the authors.

## AUTHOR CONTRIBUTIONS

C.G.T. and B.J.W. conceived and designed research; C.G.T. performed experiments; C.G.T. and K.E.N. analyzed data; C.G.T., A.E.S., K.E.N., J.S.O., R.G.F., and B.J.W. interpreted results of experiments; C.G.T. prepared figures; C.G.T. drafted manuscript; C.G.T., A.E.S., K.E.N., J.S.O., R.G.F., and B.J.W. edited and revised manuscript; C.G.T., A.E.S., K.E.N., J.S.O., R.G.F., and B.J.W. approved final version of manuscript.

## References

[1] Jonk AM, Houben AJ, de Jongh RT, Serné EH, Schaper NC, Stehouwer CD. Microvascular dysfunction in obesity: a potential mechanism in the pathogenesis of obesity-associated insulin resistance and hypertension. Physiology (Bethesda) 22: 252–260, 2007. doi:10.1152/physiol.00012.2007. 17699878

[2] Holowatz L, Thompson-Torgerson C, Kenney W. The human cutaneous circulation as a model of generalized microvascular function. J Appl Physiol (1985) 105: 370–372, 2008. doi:10.1152/japplphysiol.00858.2007. 17932300

[3] Martin Z, Shannon C, Kistler B, Nagelkir P, Del Pozzi A. Effect of sex and menstrual cycle on skin sensory nerve contribution to local heating. Int J Exerc Sci 12: 1265–1279, 2019. 31839845 10.70252/ZFHU7113 PMC6886618

[4] Stanhewicz A, Greaney J, Kenney W, Alexander L. Sex- and limb-specific differences in the nitric oxide-dependent cutaneous vasodilation in response to local heating. Am J Physiol Regul Integr Comp Physiol 307: R914–R919, 2014. doi:10.1152/ajpregu.00269.2014. 25100074 PMC4187182

[5] Patik J, Curtis B, Nasirian A, Vranish J, Fadel P, Brothers R. Sex differences in the mechanisms mediating blunted cutaneous microvascular function in young black men and women. Am J Physiol Heart Circ Physiol 315: H1063–H1071, 2018. doi:10.1152/ajpheart.00142.2018. 30074835

[6] Greaney J, Stanhewicz A, Kenney W, Alexander L. Lack of limb or sex differences in the cutaneous vascular responses to exogenous norepinephrine. J Appl Physiol (1985) 117: 1417–1423, 2014. doi:10.1152/japplphysiol.00575.2014. 25342706 PMC4269681

[7] Gagnon D, Crandall C, Kenny G. Sex differences in postsynaptic sweating and cutaneous vasodilation. J Appl Physiol (1985) 114: 394–401, 2013. doi:10.1152/japplphysiol.00877.2012. 23154992 PMC3568872

[8] Choi P, Brunt V, Fujii N, Minson C. New approach to measure cutaneous microvascular function: an improved test of NO-mediated vasodilation by thermal hyperemia. J Appl Physiol (1985) 117: 277–283, 2014. doi:10.1152/japplphysiol.01397.2013. 24903917 PMC4122693

[9] MAinson C, Berry L, Joyner M. Nitric oxide and neurally mediated regulation of skin blood flow during local heating. J Appl Physiol (1985) 91: 1619–1626, 2001. doi:10.1152/jappl.2001.91.4.1619. 11568143

[10] Williams MR, Westerman RA, Kingwell BA, Paige J, Blombery PA, Sudhir K, Komesaroff PA. Variations in endothelial function and arterial compliance during the menstrual cycle. J Clin Endocrinol Physiol 86: 5389–5395, 2001. doi:10.1210/jcem.86.11.8013. 11701712

[11] Charkoudian N, Stephens D, Pirkle K, Kosiba W, Johnson J. Influence of female reproductive hormones on local thermal control of skin blood flow. J Appl Physiol (1985) 87: 1719–1723, 1999. doi:10.1152/jappl.1999.87.5.1719. 10562614

[12] Shenouda N, Priest S, Rizzuto V, MacDonald M. Brachial artery endothelial function is stable across a menstrual and oral contraceptive pill cycle but lower in premenopausal women than in age-matched men. Am J Physiol Heart Circ Physiol 315: H366–H374, 2018. doi:10.1152/ajpheart.00102.2018. 29727219

[13] Sandoo A, Carroll D, Metsios GS, Kitas GD, Veldhuijzen van Zanten JJ. The association between microvascular and macrovascular endothelial function in patients with rheumatoid arthritis: a cross-sectional study. Arthritis Res Ther 13: R99, 2011. doi:10.1186/ar3374. 21693023 PMC3218914

[14] Ibrahimi K, De Graaf Y, Draijer R, Jan Danser A, VanDenBrink A, van den Meiracker A. Reproducibility and agreement of different non-invasive methods of endothelial function assessment. Microvasc Res 117: 50–56, 2018. doi:10.1016/j.mvr.2018.01.004. 29338981

[15] Magerl W, Treede R. Heat-evoked vasodilatation in human hair skin: axon reflexes due to low-level activity of nociceptive afferents. J Physiol 497: 837–848, 1996. doi:10.1113/jphysiol.1996.sp021814. 9003568 PMC1160979

[16] Hodges G, Pozzi AD, McGarr G, Mallette M, Cheung S. The contribution of sensory nerves to cutaneous vasodilatation of the forearm and leg to local skin heating. Eur J Appl Physiol 115: 2091–2098, 2015. doi:10.1007/s00421-015-3188-7. 25998144

[17] Sims S, Heather A. Myths and Methodologies: Reducing scientific design ambiguity in studies comparing sexes and/or menstrual cycle phases. Exp Physiol 103: 1309–1317, 2018. doi:10.1113/EP086797. 30051938

[18] Miller J, Turner C, Otis J, Sebeh Y, Hayat M, Quyyumi A, Wong BJ. Inhibition of iNOS augments cutaneous endothelial NO-dependent vasodilation in prehypertensive non-Hispanic Whites and in non-Hispanic Blacks. Am J Physiol Heart Circ Physiol 320: H190–H199, 2021. doi:10.1152/ajpheart.00644.2020. 33124886 PMC7847065

[19] Wong BJ, Turner CG, Miller JT, Walker DC, Sebeh Y, Hayat MJ, Otis JS, Quyyumi AA. Sensory nerve-mediated and nitric oxide-dependent cutaneous vasodilation in normotensive and prehypertensive non-Hispanic blacks and whites. Am J Physiol Heart Circ Physiol 319: H271–H281, 2020. doi:10.1152/ajpheart.00177.2020. 32559139 PMC7473931

[20] Turner C, Miller J, Otis J, Hayat M, Quyyumi A, Wong B. Cutaneous sensory nerve-mediated microvascular vasodilation in normotensive and prehypertensive non-Hispanic Blacks and Whites. Physiol Rep 8: e14437, 2020. doi:10.14814/phy2.14437. 32401424 PMC7219271

[21] Brothers R, Fadel P, Keller D. Racial disparities in cardiovascular disease risk: mechanisms of vascular dysfunction. Am J Physiol Heart Circ Physiol 317: H777–H789, 2019. doi:10.1152/ajpheart.00126.2019. 31397168 PMC6843015

[22] Holowatz LA, Thompson-Torgerson CS, Kenney WL. Altered mechanisms of vasodilation in aged human skin. Exerc Sport Sci Rev 35: 119–125, 2007. doi:10.1097/jes.0b013e3180a02f85. 17620930

[23] Randolph J, Sowers M, Bondarenko I, Harlow S, Luborsky J, Little R. Change in estradiol and follicle-stimulating hormone across the early menopausal transition: effects of ethnicity and age. J Clin Endocrinol Metab 89: 1555–1561, 2004. doi:10.1210/jc.2003-031183. 15070912

[24] Dillon GA, Wolf ST, Alexander LM. Nitric oxide-mediated cutaneous microvascular function is not altered in young adults following mild-to-moderate SARS CoV-2 infection. Am J Physiol Heart Circ Physiol 322: H319–H327, 2022. doi:10.1152/ajpheart.00602.2021. 34995164 PMC8803551

[25] Hodges G, Chiu C, Kosiba W, Zhao K, Johnson J. The effect of microdialysis needle trauma on cutaneous vascular responses in humans. J Appl Physiol (1985) 106: 1112–1118, 2009. doi:10.1152/japplphysiol.91508.2008. 19196910 PMC2698648

[26] Keen J, Levitt E, Hodges G, Wong B. Short-term dietary nitrate supplementation augments cutaneous vasodilatation and reduces mean arterial pressure in healthy humans. Microvasc Res 98: 48–53, 2015. doi:10.1016/j.mvr.2014.12.002. 25554360

[27] Wong B, Fieger S. Transient receptor potential vanilloid type-1 (TRPV-1) channels contribute to cutaneous thermal hyperaemia in humans. J Physiol 588: 4317–4326, 2010. doi:10.1113/jphysiol.2010.195511. 20807792 PMC3002459

[28] Wolf S, Dillon G, Alexander L, Jablonski N, Kenney W. Skin pigmentation is negatively associated with circulating vitamin D concentration and cutaneous microvascular endothelial function. Am J Physiol Heart Circ Physiol 323: 490–498, 2022. doi:10.1152/ajpheart.00309.2022. 35930446 PMC9448272

[29] Stanhewicz A, Jandu S, Santhanam L, Alexander L. Increased angiotensin II sensitivity contributes to microvascular dysfunction in women who have had preeclampsia. Hypertension 70: 382–389, 2017. doi:10.1161/HYPERTENSIONAHA.117.09386. 28652473 PMC5520653

[30] Smith C, Craighead D, Alexander L. Effects of vehicle microdialysis solutions on cutaneous vascular responses to local heating. J Appl Physiol (1985) 123: 1461–1467, 2017. doi:10.1152/japplphysiol.00498.2017. 28860170 PMC6157644

[31] Kellogg D, Zhao J, Wu Y. Endothelial nitric oxide synthase control mechanisms in the cutaneous vasculature of humans in vivo. Am J Physiol Heart Circ Physiol 295: H123–H129, 2008. doi:10.1152/ajpheart.00082.2008. 18469149 PMC2494770

[32] Cracowski J, Minson C, Salvat-Melis M, Halliwill J. Methodological issues in the assessment of skin microvascular endothelial function in humans. Trends Pharmacol Sci 27: 503–508, 2006. doi:10.1016/j.tips.2006.07.008. 16876881

[33] Cooke J, Creager M, Osmundson P, Shepherd J. Sex differences in control of cutaneous blood flow. Circulation 82: 1607–1615, 1990. doi:10.1161/01.cir.82.5.1607. 2225365

[34] Cattaneo MG, Vanetti C, Decimo I, Di Chio M, Martano G, Garrone G, Bifari F, Vicentini LM. Sex-specific eNOS activity and function in human endothelial cells. Sci Rep 7: 9612, 2017. doi:10.1038/s41598-017-10139-x. 28852041 PMC5575132

[35] Brunt V, Minson C. KCa channels and epoxyeicosatrienoic acids: major contributors to thermal hyperaemia in human skin. J Physiol 590: 3523–3534, 2012. doi:10.1113/jphysiol.2012.236398. 22674719 PMC3547267

[36] Cherney DZI, Scholey JW, Cattran DC, Kang AK, Zimpelmann J, Kennedy C, Lai V, Burns KD, Miller JA. The effect of oral contraceptives on the nitric oxide system and renal function. Am J Physiol Renal Physiol 293: F1539–F1544, 2007. doi:10.1152/ajprenal.00351.2007. 17715260

[37] John S, Jacobi J, Schlaich M, Delles C, Schmieder R. Effects of oral contraceptives on vascular endothelium in premenopausal women. Am J Obstet Gynecol 183: 28–33, 2000. doi:10.1067/mob.2000.105739. 10920304

[38] Cauci S, Buligan C, Marangone M, Francescato M. Oxidative stress in female athletes using combined oral contraceptives. Sports Med Open 2: 40, 2016. doi:10.1186/s40798-016-0064-x. 27747795 PMC5031583

[39] Cauci S, Francescato M, Curcio F. Combined oral contraceptives increase high-sensitivity C-reactive protein but not haptoglobin in female athletes. Sports Med 47: 175–185, 2017. doi:10.1007/s40279-016-0534-9. 27084393

[40] Cauci S, Xodo S, Buligan C, Colaninno C, Barbina M, Barbina G, Francescato MP. Oxidative stress is increased in combined oral contraceptives users and is positively associated with high-sensitivity C-reactive protein. Molecules 26: 1070, 2021. doi:10.3390/molecules26041070. 33670593 PMC7921945

[41] Lobysheva II, van Eeckhoudt S, Dei Zotti F, Rifahi A, Pothen L, Beauloye C, Balligand J-L. Heme-nitrosylated hemoglobin and oxidative stress in women consuming combined contraceptives. Clinical application of the EPR spectroscopy. Free Radic Biol Med 108: 524–532, 2017. doi:10.1016/j.freeradbiomed.2017.03.039. 28392282

[42] Wenner M, Taylor H, Stachenfeld N. Androgens influence microvascular dilation in PCOS through ET-A and ET-B receptors. Am J Physiol Endocrinol Physiol 305: E818–E825, 2013. doi:10.1152/ajpendo.00343.2013. 23921139 PMC3798701

[43] Hodges G, Kosiba W, Zhao K, Johnson J. The involvement of norepinephrine, neuropeptide Y, and nitric oxide in the cutaneous vasodilator response to local heating in humans. J Appl Physiol (1985) 105: 233–240, 2008. doi:10.1152/japplphysiol.90412.2008. 18483164 PMC2494831

[44] Daniels K, Abma J. Current contraceptive status among women aged 15-49: United States, 2017-2019. NCHS Data Brief 388: 1–8, 2020. 33151146

[45] Terlinden R, Uragg H, Göhler K, Kneip C. Pharmacokinetics of chlormadinone acetate following single and multiple oral dosing of chlormadinone acetate (2 mg) and ethinylestradiol (0.03 mg) and elimination and clearance of a single dose of radiolabeled chlormadinone acetate. Contraception 74: 239–244, 2006. doi:10.1016/j.contraception.2006.03.011. 16904418

[46] Baerwald A, Pierson R. Ovarian follicular development during the use of oral contraception: a review. J Obstet Gynaecol Can 26: 16–24, 2004. doi:10.1016/s1701-2163(16)30692-2. 14715122 PMC2891973

[47] Legro RS, Pauli JG, Kunselman AR, Meadows JW, Kesner JS, Zaino RJ, Demers LM, Gnatuk CL, Dodson WC. Effects of continuous versus cyclical oral contraception: a randomized controlled trial. J Clin Endocrinol Metab 93: 420–429, 2008. doi:10.1210/jc.2007-2287. 18056769 PMC2243231

[48] Kellogg D, Zhao J, Wu Y. Roles of nitric oxide synthase isoforms in cutanenous vasodilation induced by local warming of the skin and whole body heat stress in humans. J Appl Physiol (1985) 107: 1438–1444, 2009. doi:10.1152/japplphysiol.00690.2009. 19745188 PMC2777790

[49] Bruning RS, Santhanam L, Stanhewicz AE, Smith CJ, Berkowitz DE, Kenney WL, Holowatz LA. Endothelial nitric oxide synthase mediates cutaneous vasodilation during local heating and is attenuated in middle-aged human skin. J Appl Physiol (1985) 112: 2019–2026, 2012. doi:10.1152/japplphysiol.01354.2011. 22500004 PMC3378394

